# Assessment and evaluation of a serious game for teaching factual knowledge in dental education

**DOI:** 10.1186/s12909-023-04498-5

**Published:** 2023-07-20

**Authors:** Martin Lemos, Stefan Wolfart, Anne Barbara Rittich

**Affiliations:** 1 Audiovisual Media Center, Medical Faculty, RWTH Aachen University, Pauwelsstrasse 30, 52074 Aachen, Germany; 2 Department of Prosthodontics and Biomaterials, RWTH Aachen University Hospital, Pauwelsstrasse 30, 52074 Aachen, Germany

**Keywords:** Dental education, Serious game, Quiz, Factual knowledge training

## Abstract

**Objectives:**

A serious game application was developed to train factual knowledge and for self-assessment. The aim of the present study was to compare the effects of a game application (intervention group) or paper scripts (control group) on knowledge acquisition and to evaluate the acceptance of the new application among dental students.

**Methods:**

The 4th semester students of the second preclinical prosthodontics course were randomly assigned to one of the two groups (*n* = 58/51) for two consecutive years. The study was conducted in two phases: First, all participants took a pretest, with the intervention group using the game application and the control group receiving the same set of questions in a paper script. In the second phase, all participants took a post-test. After the post-test, both groups had access to the application for another three weeks. After that, all participants completed standardized questionnaires and a scale to evaluate the usability of the system. Usage statistics were also tracked. Differences between groups were evaluated together and for both years separately in terms of pretest and posttest scores and learning success.

**Results:**

There was no significant difference between the groups with regard to the posttest and learning success. A significant improvement in knowledge between pretest and posttest (*p* < 0.05) was demonstrated in both groups. Each student played approximately 350 questions. Participants rated the application with the German school grade "good". Participants appreciated the application and rated it positively. They stated that the game motivated them to learn and that they spent more time with the learning content.

**Conclusion:**

Due to the positive perception achieved through the game, this application is able to motivate students to learn. The learning effect achieved is similar to learning on paper.

## Introduction

Lack of motivation is a major challenge for globaleducation, as students with higher interest and enjoyment scores achieve better course grades [1–4]. For this reason, modern teaching must motivate students and use innovative techniques and technologies to help them achieve better results. Learning in medical education often does not ensure long-term retention [5]. Active learning methods that support students' pursuit of information are appropriate tools [1]. Methods that support recall accuracy also appear to be associated with knowledge retention [6]. Today's learners have grown up with digital technologies and should be motivated and supported by innovative learning techniques and modern technologies [7–10]. A serious game is a board game, card game, or computer game that is not exclusively invented for entertainment. Serious games – as well as educational games – have in common the concern to convey information and education in balance with entertainment aspects. Serious games can combine entertainment technology and education and are an example of active teaching–learning methods that contribute to student learning [1, 11]. Users can acquire knowledge and prepare for exams through games [12]. Motivational factors such as playfulness, fun, and ambition are combined with relevant knowledge [13]. Acquiring knowledge through play leads to an improvement in the participants' motivational range, so they spend more time learning [14–18]. Serious games are able to generate intrinsic motivation in players and achieve a high level of engagement with the learning content. When designing serious games, care must be taken to ensure that intrinsic motivation elements that are supposed to motivate do not have the opposite effect. Competition, for example, is an intrinsic motivator that can lead to stress and thus, poor performance [19]. Moreover, adding extrinsic rewards can lead to an over-justification and a loss of intrinsic motivation, especially for already motivated students. In essence, the game's primary objective contradicts the intended educational outcomes [18, 19]. The traditional linear teaching approach seems counterintuitive and can be improved by introducing serious gaming [16, 20]. Studies have shown that medical and nursing students have positive attitudes toward gaming [21–23]. Serious games are also already used in dental and medical education. Unfortunately, few studies have been conducted to examine the effect of serious gaming in dental education. However, the existing studies show a positive effect on acquiring factual knowledge and practical skills [23–29]. The types of serious games differ in terms of practical skills training, such as haptic simulators, alginate mixing simulators, or dentin bonding video games, which offer students the opportunity to practice and train theoretical content in a safe environment [25, 27, 29].

The number of smartphone users worldwide was around 3.3 billion in 2019, 300 million more than in the previous year. Data for 2020 is not yet available, but a further increase to 3.5 billion is forecast [30].

Smartphones are a central part of many students' lives [31]. Although smartphones and social media are not formally integrated into the curriculum, students are using them for their education [31, 32]. This behavior can be an opportunity for modern, innovative learning. Education with smartphones enables new approaches to learning for students by using appropriate learning methods in apps [31, 32]. The present study intended to use the described positive effects of serious gaming in combination with the existing affinity for smartphones in dental teaching and thus to increase the motivation and ambition of dental students. Another aim of this project was to gather initial experience in using and developing mobile serious games in teaching.

The present study aimed to analyze the acceptance and usage behavior of students for a newly developed serious game and to compare the knowledge gained between two groups of students, one of which used the game application and one of which did not: Central questions were, whether the learning effect of the students is higher when using the game app than when using traditional paper scripts and whether the perception of the students towards the game is positive.

By calculating the difference in scores obtained between each group's pre-test and post-test scores, we can calculate a unit for *learning success*. This factor was examined for differences between the groups IG and CG.

The null hypothesis is that there is no difference in formative post-test scores between the intervention group using the serious game and the control group using paper scripts.

## Materials and methods

### Type of study

The study was anonymous, monocentric, prospective, and comparative in design.

### App development

**Fig. 1:**
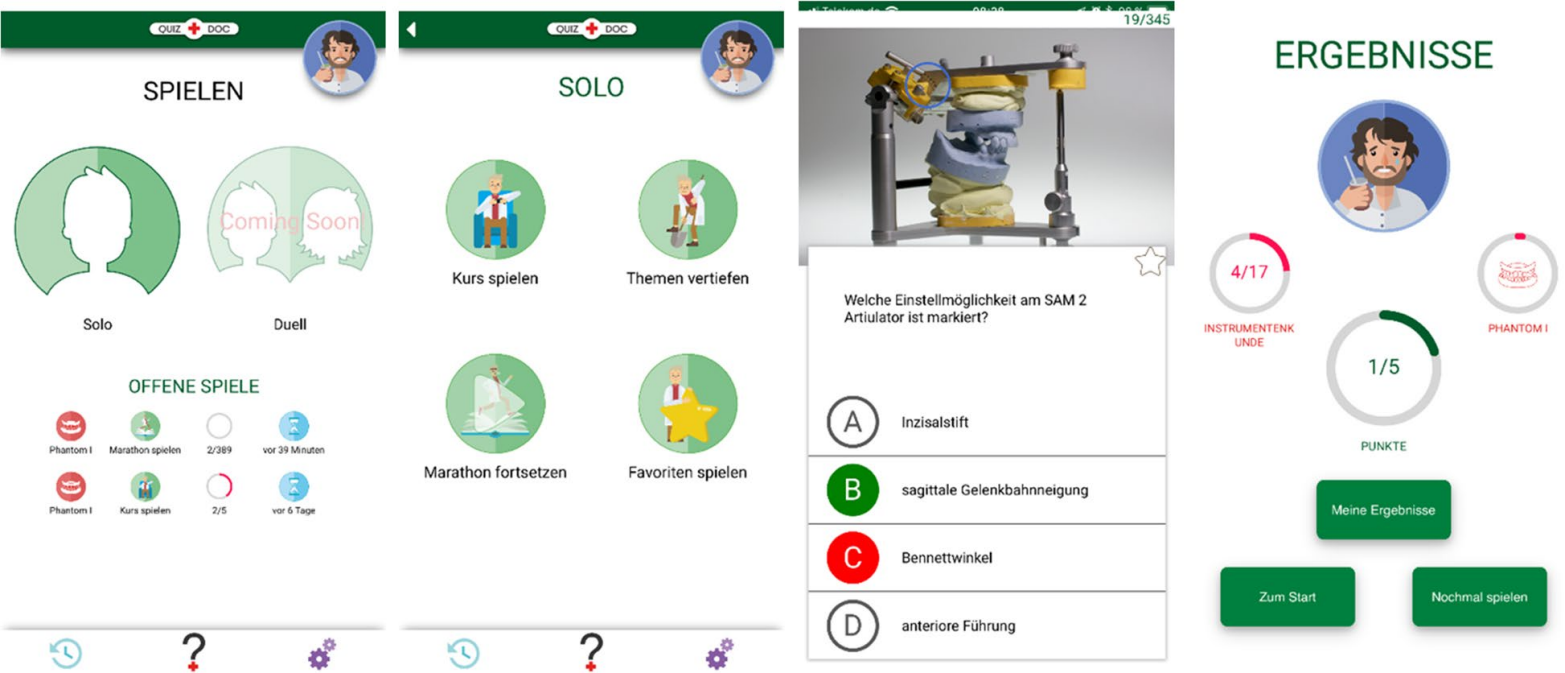
Shows screenshots of the newly programmed serious game

**Fig. 2:**
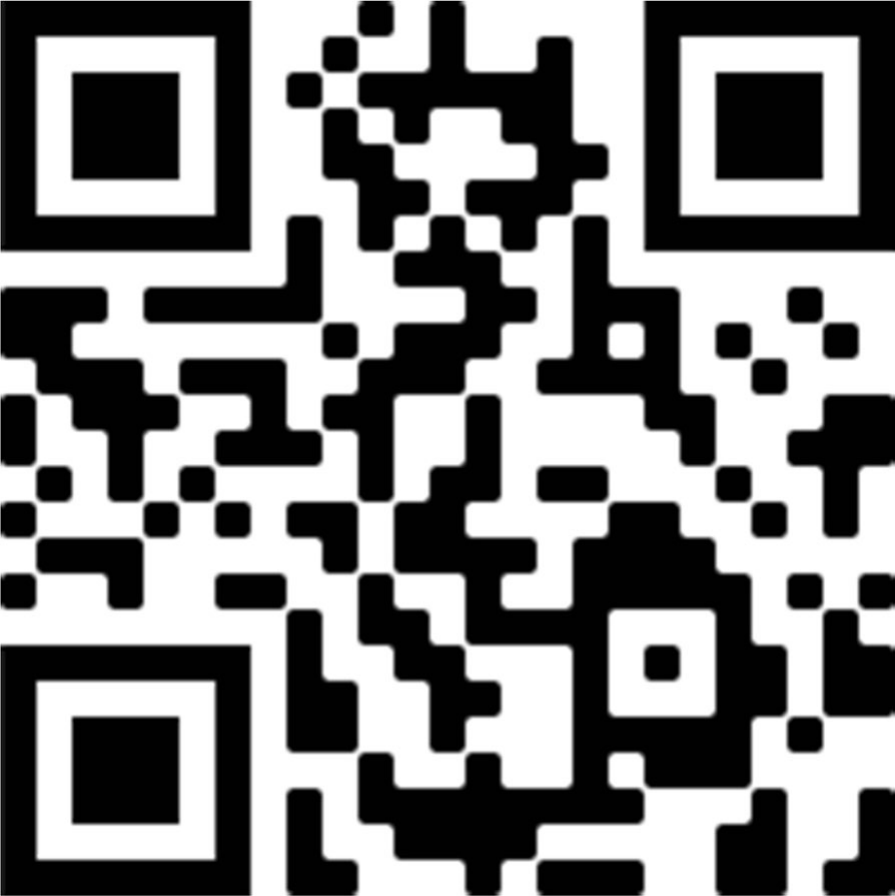
Shows a QR code linked to a video introducing the serious game app

A mobile web-app (app) for knowledge repetition and factual knowledge training was created in German and made available for dental students at RWTH Aachen University. The app is a digital quiz-based game that can be used on smartphones, tablets, and browsers on desktop computers and laptops (Figs. 1 and 2). The app was programmed, designed and successfully developed by the Audiovisual Media Center (AVMZ) of the Medical Faculty of RWTH Aachen University. The questions for the app were created by two dentists of the Department of Prosthodontics and Biomaterials and proofread by the Chair of Dental Prosthetics and Biomaterials. The initial development of the app was based on focus group interviews conducted with a group of three students, two teachers, a software developer, a media didactician and a media designer. Here, topics such as the focus of the topic, framework conditions, gamification aspects, digital platforms, and possible applications were discussed.

First, a prototype was developed, which could be optimized thanks to a beta test and further focus group interviews. The focus groups included teachers, students, and media experts. Finally, the app was finalized for this study based on the aforementioned findings and suggestions for improvement.

The app's questions are divided into categories based on the individual courses taken throughout the dental curriculum. Two dentists from the Department of Prosthodontics and Biomaterials created the questions as single-choice questions with four answer options. The chair of the department proofread all questions. The integration of graphic elements such as illustrations or photographs was provided. A game session consists of five consecutive questions. Feedback on each question played is provided after each game to promote student learning. The student plays a certain number of questions. At the end of a game, he is shown how many questions were answered correctly or incorrectly. For each correct or incorrect answer, the student receives feedback and an explanation of why the answer was correct or incorrect. Users can also create their own nicknames and choose an existing avatar. The avatar reacts differently after each game to support the gamification effect during a game session, depending on the player's performance and points scored. Depending on how many points the player has achieved, the avatar cries is neutral or laughs when he has achieved a high score and receives a reward for it. On the results page, the student also receives feedback via a round bar on how many of the questions available in the app he has answered correctly so far and how many are still missing. This is a method of tracking performance and progress in the game. The goal is to close the entire bar, and thus to have answered all possible questions correctly. The time of answering is also tracked and marked in red if the questions are answered slowly.

The app contains different modes. Participants can play all questions one after the other, regardless of the learning topic, in the so-called "Marathon" mode, or they can play questions on a specific course, in the so-called "Play Course" mode, or questions on specific topics of the course, in the so-called "Topic Deepening" mode. In addition, participants can play the "Favorites" mode, in which they can recall questions that they have previously marked as important.

The target group for the present study was the dental students of the 4th preclinical semester of the Department of Prosthodontics and Biomaterials. The entire course lasts approximately nine weeks.

The practical objectives of the course are to acquire skills in tooth preparation for fixed prostheses, fabrication of a Michigan splint, and temporary restorations.

The course is therefore divided into different practical sections. Parallel to the practical training, theoretical seminars are held once a week, and lectures twice a week.

Theory taught includes general dentistry, materials science, prosthodontics and dental instruments.

The focus of the game is on teaching detailed theoretical content, in particular learning factual knowledge such as terms, basic anatomical principles, specialist vocabulary and classifications. The lectures and seminars, on the other hand, focus mainly on manual tasks and on deepening basic theoretical knowledge.

### Participant

All students who completed the first preclinical course in dentistry were included as participants in the study. They had the same lectures, seminars, and learning/teaching materials. The study was conducted for two consecutive years. All participants of the 4th preclinical semester participated voluntarily in the study. They were randomly divided into two groups (IG = intervention group / CG = control group).

### Test procedure

Both groups completed a formative, unannounced pretest with 20 items at the beginning of the course after the first week. The theory taught included general dentistry, materials science, prosthodontics and dental instruments and were not the subject of the seminars and lectures. A total of two and zero points were awarded for each correct and incorrect answer, respectively. The maximum achievable score for each test was set at 40.

After the initial assessment, group IG received the app to train for three weeks with anonymized and randomly generated identities (IDs) and passwords. During this intervention period, group CG received the same questions as in the app as a printed booklet for self-training and self-assessment. During the test period, the app recorded usage statistics. It recorded how many questions and games of the different game modes were played and whether the answers given were correct or incorrect. After the intervention phase, both groups participated in a formative, unannounced post-test with 20 tasks similar to those of the pretest. After the post-test, the CG groups had access to the app for three more weeks with anonymized and randomly generated IDs and passwords. At the end of the course, both groups completed standardized questionnaires to evaluate the app. The items consisted of a six-point Likert scale, with extremes of "strongly agree" and "strongly disagree". Additionally, participants were asked in which situation and how often they played with the app and which German school grade they would rate the app. The app's usability was tested using the System Usability Scale (SUS) [33–35]. The SUS provides a reliable tool for measuring the usability of products and services, including hardware, software, mobile devices, websites and applications. It consists of the following 10-item questionnaire with five response options for respondents, from strongly agree to strongly disagree [33–35]:

- I think that I would like to use this system frequently.
- I found the system unnecessarily complex.
- I thought the system was easy to use.
- I think that I would need the support of a technical person to be able to use this system.
- I found the various functions in this system were well integrated.
- I thought there was too much inconsistency in this system.
- I would imagine that most people would learn to use this system very quickly.
- I found the system very cumbersome to use.
- I felt very confident using the system.
- I needed to learn a lot of things before I could get going with this system.

### Statistics

Statistical analysis was performed using SPSS Statistics 25 software (IBM).

By calculating the difference in scores obtained between pre-test and post-test scores of each group, we can calculate a unit for *learning success*. This factor was examined for differences between the groups IG and CG.

Descriptive statistics were used to summarize game use and assessment results. For the analysis of differences between groups in terms of pre-test/post-test, the Wilcoxon Signed Rank Test was used. For the analysis of differences between groups in terms of pre-test/post-test scores and differences between pre-test and post-test, the Mann–Whitney test was performed. Significance was assumed at *p* < 0.05.

## Results

### Participant

The study was conducted in two consecutive years. All 4th semester preclinical students of two consecutive years (*n* = 58 / 51 = 109) volunteered as participants in the study. The IG consisted of 55 (25 in the first year / 30 in the second year) and the CG of 54 (26 in the first year / 28 in the second year) students.

### Pre-/post-test

First, the results of the two semesters were evaluated individually.

**Fig. 3:**
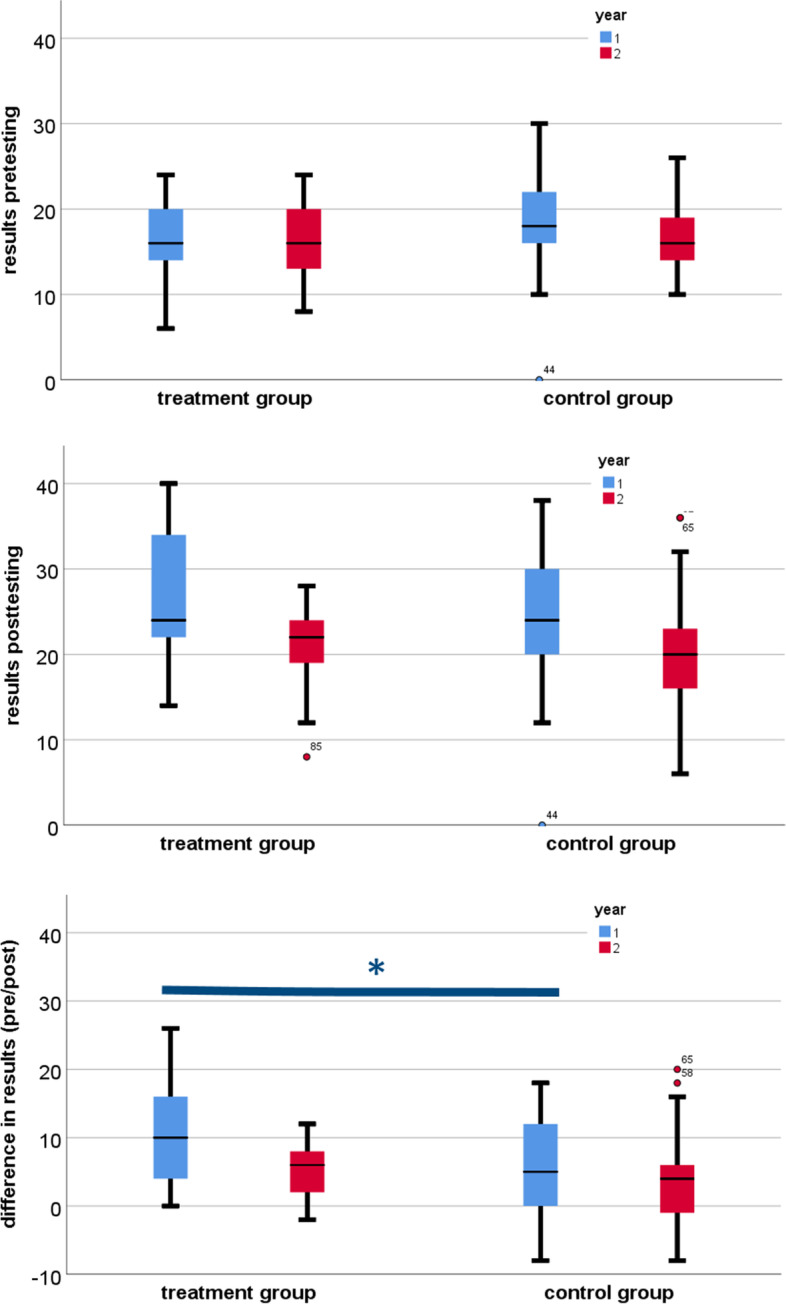
Shows the pre-test results (first diagram), post-test results (second diagram) and the differences between the pre-test and post-test results (last diagram). The first year's results are marked in blue and those of the second year in red. The maximum number of points achieved was 40. The star indicates the significant difference

In both years, there was a significant difference between the pre-test and post-test for IG and CG (*p* < 0.05). Both groups scored better on the post-test than on the pre-test (Fig. 3).

There was no significant difference between IG and CG in terms of pre-test and post-test scores in both years.

After calculating the difference in scores obtained between the pre-test and post-test scores of each group to obtain a unit for *learning success*, this factor was examined for differences between the groups IG and CG. There was a significant difference in learning success in the first year (*p* < 0.05) and in the second year, there was almost a significant difference as the p-value was exactly 0.05 between IG and CG. In the first year, the intervention group had better results in learning success than the control group (Fig. 3).

**Fig. 4:**
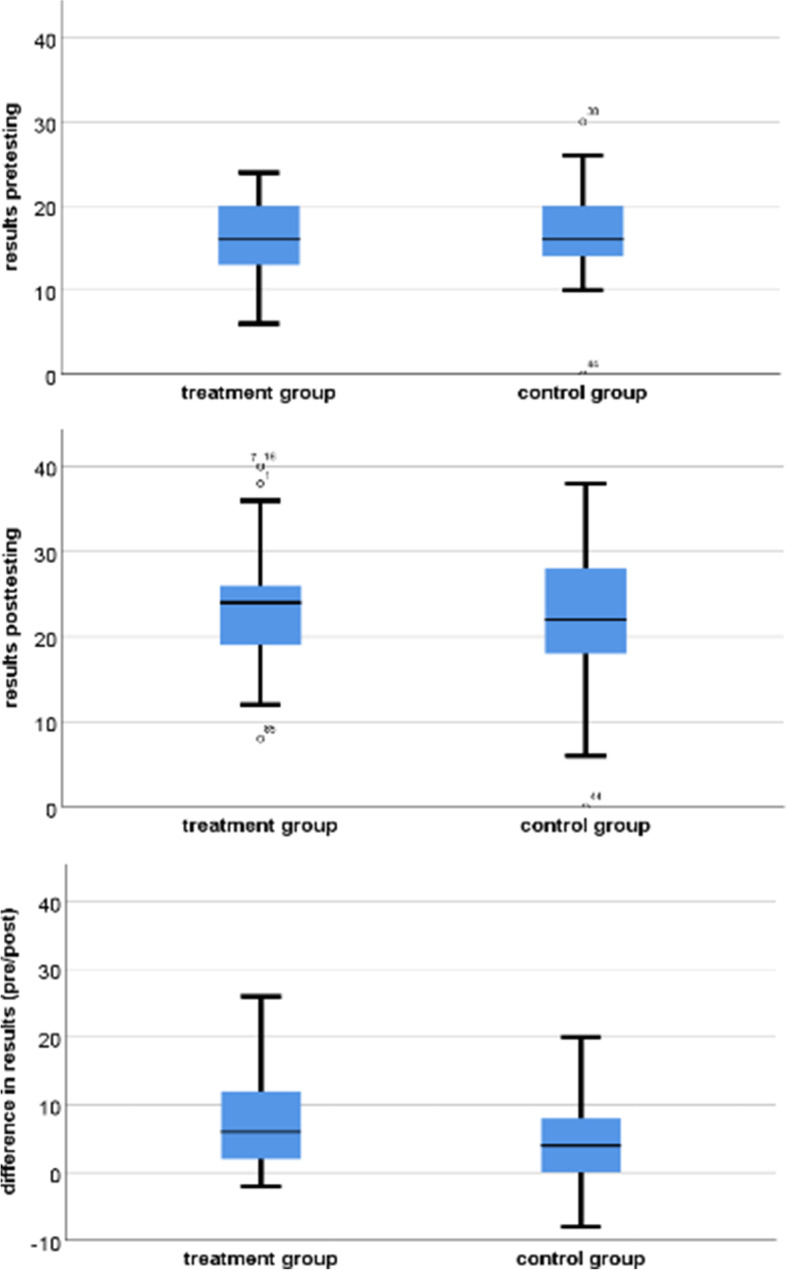
Shows the pooled results of the first and second year

Analysis of pooled results for both years showed no significant difference between the two groups in pre-test and post-test scores and learning success. Also, in the pooled data analysis, the mean values of post-test scores and learning success for IG were higher than in the individual years analysis (Fig. 4).

### Evaluation and usage statistics

Of the 109 participants, 87 completed the evaluation forms.

**Fig. 5:**
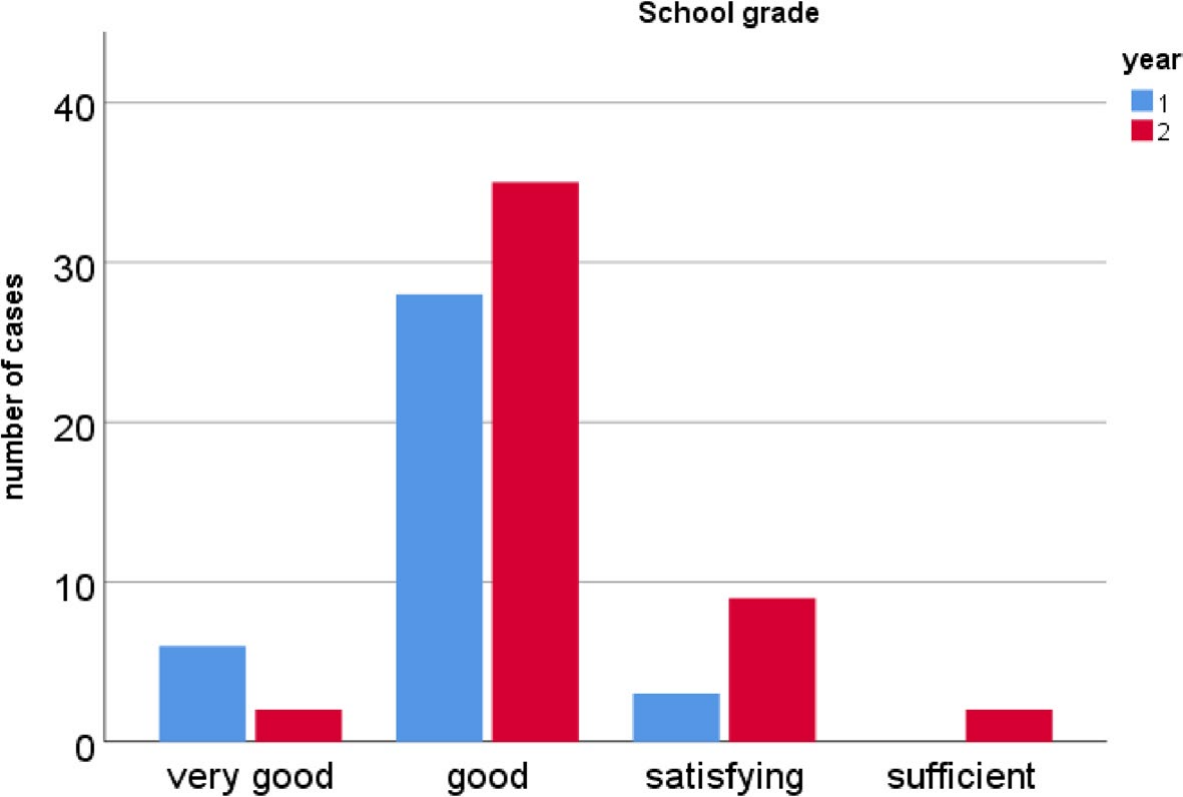
Shows the evaluation results concerning the statement "I give the app the German school grade". The first year's results are marked in blue and those of the second year in red

Most participants would use the app regularly (78.2%). They also liked the design (73%), thought it was easy to use (85%), and would recommend the app to others (> 85%). Ninety-seven percent of participants thought learning with the app was fun. Most participants (89%) said the app motivated them to learn. Most participants said the app helped them learn factual knowledge faster (89%). Eighty-two percent of participants said they spent more time studying/learning material because of the app. Whether the app prepared them well for the exam was answered inconsistently by the participants: 52% answered rather positively and 48% rather negatively. 10% answered that they did not use the app. Most participants (84%) felt the app did not distract them from learning. Responses to the question whether they played longer than they wanted to were mixed. For example, 37.6% answered rather positively. Most participants rated the feedback feature as helpful (68%) (Table 1). Most participants in both years rated the app as "good" (74%) and as many as 9% rated it as very good (Fig. 5).

**Table 1:** Examples of results from questionnaires summarized for all participants are part of the SUS (^a^) with a 5-point Likert scale (1= strongly agree to 5 = strongly disagree). Additional questions were scored using a 6-point Likert scale. (1 = strongly agree to 6 = strongly disagree).

	**N**	**mean score**	**Standard deviation**
I can very well imagine using the app regularly^a^	87	1.90	0.86
I think the app is unnecessarily complex^a^	87	4.32	0.86
I think the app is easy to use^a^	87	1.70	0.67
I think the app is self-explanatory^a^	87	1.56	0.68
I find the design very appealing^a^	87	2.09	0.83
I would recommend the app	87	1.79	0.82
Learning with the app is fun	87	2.10	0.90
The app motivates learning	87	2.30	1.02
Thanks to the app I was able to learn facts faster	87	2.22	1.07
The app has made me more engaged with learning content	87	2.47	1.17
The app distracted me from learning	86	3.59	1.14
I did not use the app for learning	87	4.59	1.27
I have played longer than I wanted	87	4.31	1.48
The feedback function was helpful	85	3.65	1.39
I rate the app with German school grade	81	2.44	1.34

The obtained SUS value is 77.70 (σ = 12.28, median = 77.5) (Table 1).

Tracking data provided that participants played the game at varying intervals. Overall, 6.9% of participants played several times a day, 9.9% played once a day, 28.7% played several times a week, 22.8% played once a week, 13.9% played less than once a week with the app, and 14.9% did not use the app.

During the study period, a total of 38,263 questions and 1,724 games were played by all participants. Although both groups played almost the same number of questions, the second year answered more than twice as many questions incorrectly (Table 2).

**Table 2:** Game statistics for all participants of IG and CG

	first year (*n* = 58)		second year (*n* = 51)		Both years
	Total number	Median	Total number	Median	Total number
questions played	19,816	341.7	18,447	361.5	38,263
correct questions	15,180	261.7	7,876	154.4	23,056
wrong questions	4,636	79.9	10,571	207.3	15,207
games	789	13.6	935	18.3	1,724
"course" games	229	4.0	191	3.7	420
"theme" games	344	6.0	682	13.4	1,026
"marathon" games	69	1.2	37	0.7	106

## Discussion

In both groups, there was a significant difference between the results of the pre-test and the post-test. This difference was to be expected since all participants had taken part in continuing education during the course. This result reflects the increase in knowledge during the course period. The IG who used the game app had increased knowledge, as did the CG, proving that learning with the serious game seems to have a positive effect as the conservative scripted learning that supports the general course content such as lectures and seminars. However, we cannot measure the effect of the scripts and the game because all students also attended lectures and seminars which also have an impact on the learning effect.

In both years, there was no significant difference between the two groups' data on the pre-test. It appears that both groups had similar pretest knowledge. There was also no significant difference between the two groups on the post-test results in either year. Based on these results, the null hypothesis is accepted. Both groups appear to be similarly prepared for the post-test. However, it also shows that learning with the app performs as well as learning with the paper scripts, as mentioned earlier. The results are consistent with the literature [15]. One study even reports that gamification can improve and retain knowledge better than traditional lectures [36].

However, the significant difference in the first year (*p*< 0.05) in terms of score gain between pre-test and post-test, the so-called learning success, suggests that the learning progress of the test group might be higher than that of the control group. The IG had a higher average median regarding learning success in both years and also in the pooled analysis of both years together. The quiz app seems to have had a positive effect on participants' learning success compared to the booklets in first year and was not inferior to the booklets with regard to learning success in second year and pooled data. One reason for this effect in first year could be that the app users were more motivated and answered more questions about the training, as has been reported in the literature [14–17, 23]. To substantiate this claim, however, the time that the students in the control group used to learn with booklets should also have to be measured and compared to IG. Both forms of learning seem to be at least equal in terms of learning effect. In medical education, learning basic factual knowledge is often unsupported and underappreciated. Applying learning concepts to smartphones can significantly improve the learning of factual knowledge [37]. Because of the fact that factual knowledge is difficult to learn for students, we see the motivation and fun factor as a huge advantage over traditional teaching methods. This is supported by the statement that the participants felt that they could learn facts faster with the app and that they spent more time on the learning/teaching material because of the app, but in contrast, the app did not distract them from learning. Some students even answered that they spent more time with the app than they wanted to. Our findings are consistent with the literature mentioned at the beginning of this paper, which also characterizes serious games as a motivator for students to improve their learning experience [14–17, 23]. Unfortunately, we cannot support this thesis with facts, as it is not possible to measure how many questions CG answered using paper scripts, as we were not able to objectively measure their self-study using the scripts.

Van Gaalen et al. (2021) describe that increased use of learning materials generally indicates repetition. This is one of the strongest variables affecting memory, leading to better learning outcomes and retention [38]. They note that students do not need to be distracted from repetition and reinforcement by game elements such as competition. Taveira-Gomes et al. (2015) reported that quizzes alone led to a modest increase in recall accuracy and that the study quiz task had a high impact on recall accuracy in their study [6]. The quiz structure of our app could also have a positive effect on recall accuracy.

What is striking about the data is that the second cohort shows poorer results than the first cohort in both the pre- and post-tests. They appear to represent a weaker learning group, but their results show similar learning trends to those of the first group regardless of learning type (CG or IG).

The participants enjoyed using the app and had fun learning. They rated it as good and motivating. This seems to be the main benefit of implementing serious gaming. However, it must be kept in mind that rewarding a previously unrewarding activity such as learning factual knowledge, can lead to a shift from intrinsic to extrinsic motivation [38]. This can lead to decreased interest in the activity when the rewards are no longer given. This is referred to as the "overjustification effect" [38]. However, a study reports that using Serious Games can reduce test anxiety and that learning with these games is more fun than traditional instruction [12, 15, 23]. The SUS in our study correlates with the good to excellent scale, which is higher than the mean (70.14) and has a Cronbach's alpha of 0.91 [33–35]. These results align with the literature that reports high acceptance of serious gaming by students and mentions it as a motivating factor [14, 15, 22, 23, 36, 39]. Due to the study design, however, we cannot say whether the students in CG were not just as motivated as the students in the test group, since we could not collect comparable values to SUS in CG. In contrast, however, there is also a study showing that students' satisfaction with serious gaming is modest and students' willingness to use gamification is low [40].

The positive evaluation of the students in our study may be due to the fact that the students were involved in the creation of the app and their wishes and requirements were taken into account during the development. The app was optimized for their needs. Particular attention was paid to simple, intuitive operations with an appropriate amount of information that does not overwhelm the students. This perception is also supported by Aboalshamat et al. (2020), who suggests in a study that the amount of information and its complexity are important factors in achieving the desired improvement through gamification [36]. However, the good evaluation results must also be viewed critically from the perspective of a novelty effect.

The participants stated that they did not feel well-prepared by the app for the final exams relevant to their studies. One reason could be that students have theoretical and practical exams, and thus not only pure factual knowledge but also application tasks are tested. We should have differentiated this question into practical and theoretical exams since the app only trains the theory of low taxonomy. We should also consider improving the question format in the app so that students feel prepared for questions with higher taxonomy.

The number of games and questions played are proof of the app's acceptance. On average, each student played 351 quiz questions. However, we know from the evaluation that some participants did not use the app at all (*n* = 16). Consequently, 93 students used the app, corresponding to an average of 411 questions per student. Unfortunately, we cannot extrapolate these results to the control group because we do not know how often they used the paper scripts.

Based on the evaluation data, we can assume that the gaming behavior of the participants was very different. On the one hand, there seem to be enthusiastic players who used the app at least once a day (15%) and on the other hand, more than half of the participants played at least once a week (51.5%). This shows that the participants have different needs, and the app seems unsuitable for everyone as a learning medium. Why some participants did not use the app could not be explained from the evaluation. One reason could be that the control group received the app late in the course and they were already in the learning phase for the course-relevant exams and therefore did not want to switch to the app. This statement was at least noted on isolated evaluation forms and is therefore only a thesis and not proven. Another reason could be that these students generally do not like to play with their cell phones or prefer to learn in other ways. A query about mobile gaming behavior in the evaluation questionnaire would have been helpful for interpretation.

As for books, the literature shows that some students prefer to learn with books rather than ebooks or other digital alternatives. A University of California study showed that most 390 students still prefer to read their academic texts in print if they want to achieve a deep learning outcome [41]. Some students cite the risk of distraction and poorer text comprehension as classic problems of digital learning. Many readers learn content by remembering where it is printed in the book. They think underlining and taking notes was also easier with printed paper [41]. For some students, paper-based learning materials for learning in context are seemingly more attractive than digital formats.

The strength of our study is its design as a randomized intervention trial. The knowledge level of both groups was assessed before the intervention. The app was studied in different phases. Not only was student acceptance determined, but objective testing was conducted, usage data was tracked, and the validated System Usability Scale was used to assess the app's utility in detail.

The limitations of the current study are the small number of cases, the untraceable use of paper scripts, and the lack of comparison with exams relevant to the study. Our study only shows the results of a small number of students over two consecutive years at one site. This is not generally representative, so further studies large numbers of students should be conducted in a multicenter design. Due to rigorous privacy data regulations, we were unable to correlate app usage data with study-relevant exam scores. It was also not possible to record the use of the paper scripts or this could only have been done based on the students' statements, which led to an enormous bias in the actual tracking of the app usage data and thus would not have yielded any reliable statements. In addition, pre-test and post-test were not mandatory for passing the course and were unannounced, which could imply a lower level of engagement in preparing for the tests.

We would like to mention that it is essential to involve all groups in developing and producing an application to make it a success. It is important to create the necessary personnel and technical conditions and to keep an eye on the cost–benefit ratio. If these prerequisites are met, we can only recommend implementing such a project.

The use of the game has achieved a high level of acceptance among students and teachers. Therefore, further technical developments and an expansion to other courses and faculties are planned. In addition, an implementation of new question types, the integration of video data and questions of higher taxonomy and game modes such as "duel" will take place and the possibility of creating questions by students themselves will be integrated. Perhaps we could achieve even better results with the app if the gamification character of the app were strengthened. In particular, competition and scoring seem to positively impact learning [38]. Further research projects will examine the success of the app with a higher number of students, the competitive effect of a duel mode in which players compete against each other, and the success of integrated learning groups within the game and their influence on extrinsic and intrinsic motivation. The influence of rewards, so-called badges and rankings within the game, will also be the subject of future studies. A comparison not only with paper media but also with digital media such as online PDFs or podcasts would also be desirable. Especially in times when digital teaching is becoming more and more important, the use of serious gaming is an opportunity to improve teaching.

## Conclusion

Due to the positive perception through the game, this app is able to motivate students to learn and does not interfere with learning. The learning effect achieved is similar to learning on paper.

From our results, it can be concluded that game-based learning successfully acquired theoretical skills.

## Data Availability

The datasets used and/or analyzed during the current study are available from the corresponding author on reasonable request. All data generated or analyzed during this study are included in this published article.
